# Safety and superior immunogenicity of heterologous boosting with an RBD-based SARS-CoV-2 mRNA vaccine in Chinese adults

**DOI:** 10.1038/s41422-022-00681-3

**Published:** 2022-06-14

**Authors:** Xiaoqiang Liu, Yuhua Li, Zhongfang Wang, Shouchun Cao, Weijin Huang, Lin Yuan, Yi-Jiao Huang, Yan Zheng, Jingjing Chen, Bo Ying, Zuoyun Xiang, Jin Shi, Jincun Zhao, Zhen Huang, Cheng-Feng Qin

**Affiliations:** 1 Yunnan Province Centre for Disease Control and Prevention, Kunming, Yunnan China; 2 National Institutes for Food and Drug Control, Beijing, China; 3 Respiratory Medicine, Guangzhou Institute of Respiratory Health, Guangzhou, Guangdong China; 4 Walvax Biotechnology Co., Ltd., Kunming, Yunnan China; 5 State Key Laboratory of Pathogen and Biosecurity, Beijing Institute of Microbiology and Epidemiology, Academy of Military Medical Sciences, Beijing, China; 6 Suzhou Abogen Biosciences Co., Ltd, Suzhou, Jiangsu China; 7 Research Unit of Discovery and Tracing of Natural Focus Diseases, Chinese Academy of Medical Sciences, Beijing, China

**Keywords:** Immunology, Biological techniques, Cell biology

Dear Editor,

To May 2022, the COVID-19 pandemic has claimed more than 6.28 million lives, with more than 524 million confirmed cases worldwide. The recent emergence of highly transmissible Omicron variant of severe acute respiratory syndrome coronavirus 2 (SARS-CoV-2) has triggered another major surge in both confirmed cases and deaths.^1^ Ten COVID-19 vaccines have been approved by the World Health Organization (WHO) for emergency use, including the two mRNA vaccines, BNT162b2 and mRNA-1273, and two Chinese inactivated vaccines, CoronaVac and BBIBP-CorV. However, the rapid waning of vaccine-induced virus-neutralizing antibody titers and the continuous emergence of variants of concern (VOCs), including Alpha, Beta, Delta and Omicron, have created unprecedented challenges in the eradication of COVID-19 pandemic.^2–4^

Especially, the heavily mutated Omicron variant has been well characterized to escape from most therapeutic monoclonal antibodies, as well as sera from convalescent patients or fully vaccinated individuals.^5,6^ Recent work indicated that neutralizing antibody against Omicron was absent or undetectable in most Chinese populations who received two-dose inactivated vaccines,^7^ while a booster dose with mRNA vaccine BNT162b2 showed significant superiority over homologous booster in protection against Omicron.^7–9^ However, the two commercial mRNA vaccines, encoding the full spike (S) protein of SARS-CoV-2, are not available in mainland China. The “made-in-China” mRNA vaccine candidate AWcorna (originally termed ARCoV), which encodes the receptor binding domain (RBD) of SARS-CoV-2 S protein, is being tested in the final stage of multiple-center phase III trials (https://clinicaltrials.gov/ct2/show/NCT04847102). It is of highly priority and urgency to provide evidence to support a better boosting strategy in mainland China for decision maker.

Herein, we reported the safety and immunogenicity of a third dose of heterologous boosting with AWcorna in Chinese adults who have received two-dose inactivated vaccines. The randomized clinical trial (ChiCTR2100053701) enrolled 300 adults (ages ≥ 18 years). All eligible subjects received 2-dose priming vaccination with the inactivated vaccine, CoronaVac or BBIBP-CorV. At about 6-month post-priming, all subjects were randomly assigned to either the AWcorna (*n* = 200; heterologous) or CoronaVac (*n* = 100; homologous) booster group (Supplementary information, Fig. S1a). In the AWcorna group, the median age was 43.0 years (Interquartile rate, IQR: 36.5–49.0), and the CoronaVac group was 40.0 years (IQR: 34.0–48.5) (*P* = 0.5165) (Supplementary information, Table S1). There were 116 (58%) and 55 (55%) male participants in the AWcorna and CoronaVac groups, respectively (*P* = 0.6208). Meanwhile, no significant differences were observed in the Body Mass Index (BMI), vital signs, and comorbid condition between the two groups at the baseline (all *P* > 0.05). All subjects completed the enrollment vaccination and three blood examinations consecutively at pre-booster or 0 days, 14 ± 2 days, and 28 ± 2 days post-booster vaccination. Subsequently, the neutralization and IgG antibody titers against wild-type (WT) SARS-CoV-2 and VOCs were assessed at pre-booster, 14- and 28-day post-booster by the standard cytopathic effect (CPE)-based assay and ELISA, respectively (Supplementary information). The WHO standard IgG antibody (NIBSC code 20/136) was used as a reference sample for all serological assays.

As expected, the live virus neutralization titers against WT SARS-CoV-2 were below the detection limit before boosting in all participants from both groups (Fig. 1a). Remarkably, AWcorna booster induced a 66.2-fold increase against WT SARS-CoV-2, and the geometric mean titers (GMTs) reached 293.9 and 242.4 at 14 and 28 days post booster, respectively (WHO Reference cut-off 1:139), while the GMTs in CoronaVac booster groups was only 89.1 and 64.3, respectively (Fig. 1a; Supplementary information, Table S2). Similarly, the neutralization antibody titers against the Delta variant also increased significantly after the third dose booster either with AWcorna and CoronaVac, while the GMTs in Awcorna groups were 5.1- and 6.5-fold higher than those in CoronaVac group at 14 and 28 days post booster (Fig. 1b; Supplementary information, Table S2). In addition, the increasing trends are similar in both 18–59 and ≥60 years old participants (Supplementary information, Fig. S2a, b).

**Fig. 1:**
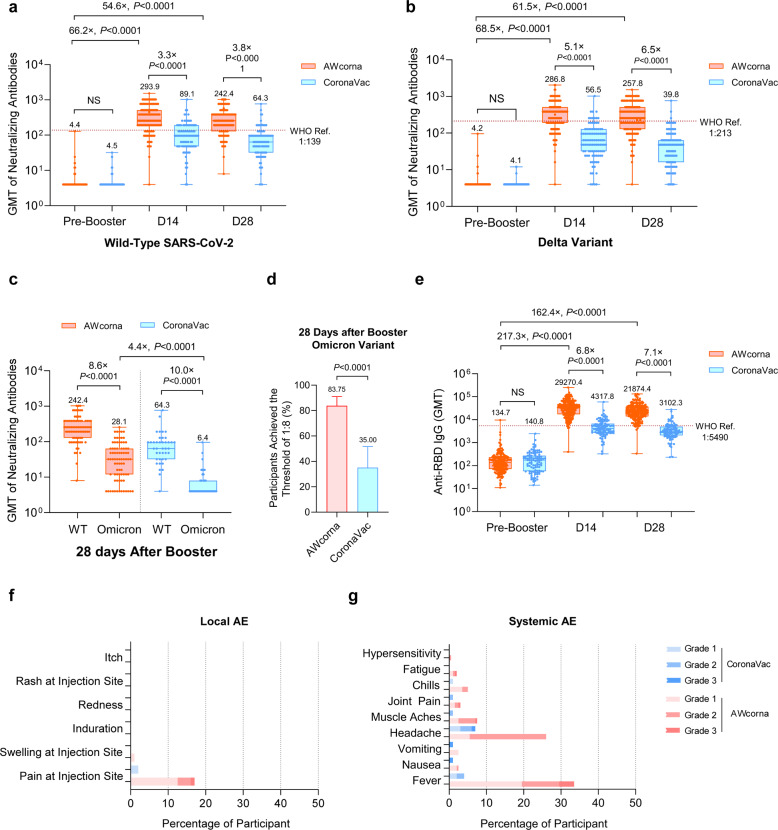
Safety and immunogenicity of heterologous boosting with AWcorna in Chinese adults. **a** Geometric mean titer (GMT) of neutralizing antibodies to live WT SARS-CoV-2. The WHO reference serum (1000 IU/mL) was equivalent to a live viral neutralizing antibody titer of 1:139 against WT. **b** GMT of neutralizing antibodies to the Delta variant. The WHO reference was 1:213 against the Delta variant (the dash line in red). Eligible participants primed with 2 doses of inactivated vaccine were randomly allocated to AWcorna group (*n* = 200) and CoronaVac group (*n* = 100) to receive a booster dose. **c** GMT of neutralizing antibodies to the Omicron variant was measured in the subgroup (120 participants with the first 120 subject numbers, 80 from AWcorna and 40 from CoronaVac group). Sera were measured 28 days after booster only, and thus no baseline analysis was performed. **d** Seropositive rates (%) of neutralizing antibody to the Omicron variant. **e** GMTs of anti-RBD IgG antibodies to WT SARS-CoV-2. The WHO reference (1000 binding antibody unit (BAU)/mL in serum) is equivalent to an RBD-specific IgG ELISA antibody titer of 1:5490. The cutoff value for the response was 1:8 for live virus neutralizing antibody and 1:10 for anti-RBD IgG. **f** The percentage of participants with local adverse events (AEs). **g** The percentage of participants with systemic AEs. These AEs were monitored in the 14-day window after the administration of the booster. For **a**–**c** and **e**, GMT data are presented in box-and-whisker plots. The figures above error bars indicate the percentage. *P* values were obtained from comparisons between the two treatment groups using *t*-tests for log-transformed antibody or two-sided χ^2^ tests for categorical data (**a**–**e**).

Despite the neutralization antibody titers against the Omicron variant showed significant reduction in comparison with those against WT in both groups, the GMTs against Omicron maintained 28.1 at 28-day after AWcorna booster, while the GMT in the CoronaVac booster group was only 6.4 (Fig. 1c; Supplementary information, Table S2). Most importantly, 83.75% of participants in the AWcorna booster group achieved the 1:8 threshold of neutralization antibody titers against the Omicron compared to only 35% of participants in the CoronaVac booster group (Fig. 1d; 95% Confidence Interval, CI: 30.82–63.84; *P* < 0.0001). Moreover, the RBD-specific IgG antibodies titers also showed a sharp increase in both booster groups, and the GMTs in AWcorna booster group were 6.8- and 7.1-fold higher than those in the CoronaVac booster group at both 14-day and 28-day time points, respectively (all *P* < 0.0001) (Fig. 1e; Supplementary information, Table S3). Taken together, these results demonstrate that heterologous boosting with AWcorna induces higher neutralization and IgG antibodies against WT, Delta and Omicron variants than homologous booster.

Additionally, we observed the safety profile of the booster dose of AWcorna. Solicited local and systemic adverse events (AEs) were recorded within 30 min and in a window of 0–14 days, and unsolicited AEs were documented within 0–28 days post-booster vaccination (Supplementary information, Table S4). For both vaccines, pain at the injection site is the most reported local AE (incidence rate, IR: 17% in AWcorna vs 2% in CoronaVac; *P* < 0.0001), mostly at the Grade 1 level (Fig. 1f). Fever was the most common systemic AE (IR: 33.5%), followed by headache (IR: 26.0%) and muscle aches (IR: 7.5%) in AWcorna group (Fig. 1g). A total of 8 subjects reported grade 3 fever (IR: 4%) among the 200 participants in AWcorna group. For the CoronaVac group, headache represented the most frequent systemic AE (IR: 7.0%), followed by fever (IR: 4.0%). No serious adverse events (SAEs) were reported in both groups.

Collectively, our present study clearly demonstrated that a 3rd dose of heterologous boosting with AWcorna was safe and protective against the circulating Delta and Omicron variants. Compared with phase 1 trial, the total IRs of local and systemic AEs for AWcorna booster showed significant improvement,^10^ especially the IR of grade 3 fever that was reduced to 4% (Supplementary information, Table S4), comparable to the other two approved mRNA vaccines.^11^ The phase 1 trial of AWcorna only included 20 adults aged 18–59 (15 μg group), while our present cohort enrolled 200 participants, including 10 subjects aged over 60. The expansion of sample size and improvement in vaccine manufacturing technologies contributed to the improved safety profile observed in our present study. The ongoing international phase 3 trials with 28,000 participants will provide more about the safety profile of AWcorna.

Previously, we have demonstrated that homologous boosting with AWcorna readily induced high neutralization antibodies against WT and Omicron variant in mice.^12^ and our present finding in human further supported heterologous booster with this China-made mRNA vaccine AWcorna in Chinese populations. Many cities in China are under the attack of Delta and Omicron variants, while few or no neuralization antibodies against Omicron were detected in most Chinese populations.^7,13^ A third dose booster has been recommended by the WHO and National Health Commission of China. Of all COVID-19 vaccines generated from different technology platforms, mRNA vaccine represent the most reasonable choice as either homologous or heterologous booster. The neutralization titers against Delta and Omicron variants in AWcorna booster group were 6.5-fold and 4.4-fold higher than those in CoronaVac booster group, respectively (Fig. 1b, c), and the AWcorna booster induced the seroconversion of Omicron neutralization in over 83% individuals (Fig. 1d). A third dose of S-targeting mRNA vaccine was evidenced to increase the number of RBD-specific memory B cells with expanded potency and breadth, thus contributing to the additional protection against VOCs including Omicron,^14,15^ highlighting the rationale of RBD-targeting mRNA vaccine as a booster. Given that Chinese population who have received three-dose inactivated vaccines is growing, additional clinical trials are being conducted to assay the benefits of heterologous boosting with AWcorna.

Finally, despite the vaccine effectiveness of AWcorna booster in preventing infection by SARS-CoV-2 and other VOCs remains to be determined, the induction of potent neutralization antibodies against WT and VOCs, as well as the affordable safety profile, support the emergency use of AWcorna as heterologous booster in China. A more potent mRNA vaccine and improved booster strategy should be warranted to meet the urgent and huge need to stop the ongoing Omicron outbreaks in China and COVID-19 pandemic worldwide.

## Supplementary information

Supplementary Information
