# Supplementary material for: Safety and superior immunogenicity of heterologous boosting with an RBD-based SARS-CoV-2 mRNA vaccine in Chinese adults
Source: Cell Res. 2022 Jun 14;32(8):777–80. doi: 10.1038/s41422-022-00681-3 (PMC9197092; doi:10.1038/s41422-022-00681-3)
Supplement: Supplementary file 1 — Supplementary Information [file 41422_2022_681_MOESM1_ESM.pdf]

Supplementary information

Methods and materials

Study design

We conducted a randomized clinical trial involving 300 adults (≥18 years of age) who were tested negative by RT-PCR screening for COVID-19 at the time of participation to elucidate the immunogenicity and safety of an mRNA-based vaccine (AWcorna) as a booster compared to that of homologous booster using an inactivated viral vaccine (CoronaVac).

Ethics statement

The trial was reviewed and approved by the Research Ethics Committee of the Center for Disease Control and Prevention of Yunnan province. The study protocol and related materials were approved by the independent Ethics Committees as well, and this trial was conducted in accordance with the Declaration of Helsinki and Good Clinical Practice with the register number of ChiCTR2100053701 (NO. 2021-15). Written informed consents were obtained from each participant before the screening.

Participants

Eligible participants met all inclusion criteria and did not trigger any exclusion criteria. Those aged 18 years and above, received full 2-dose inactivated viral priming around 6 months ago. Of the 300 participants, 175 received 2-dose CoronaVac, 14 received 2-dose BBIBP CorV, and 111 received one-dose CoronaVac and 1-dose BBIBP CorV. Participants with a previous clinical or virologic COVID-19 diagnosis or SARS-CoV-2 infection or women with positive urine

pregnancy test results were excluded from this study. Participants with a medical history of convulsion, serious acute hypersensitive reaction to vaccines, acute febrile diseases or infectious diseases, congenital or acquired angioedema, asplenia or functional asplenia, thrombocytopenia or other coagulation disorders, anti-allergy therapy, or blood products within 3 months were also excluded.

Randomization

Each participant was assigned a unique subject ID by authorized assigners successively according to a Prespecified allocation kit, which was generated by an independent randomization statistician from Beijing Key Tech Statistical Consulting Co., Ltd. via SAS software (SAS® Institute, Cary, North Carolina, USA) with the ratio of 2:1 to the AWcorna and CoronaVac groups. Since the different appearances of the two kinds of vaccines, inoculators could not keep in blind when vaccines had been used. And hence, staff who were assigned to inoculate would not be involved in any other research jobs, especially for subjects' safety follow-up procedures. Participants would be masked when receiving the jab by a special curtain in the injection room to avoid the identification of he or she, and disclosure of the allocated group. Other investigators, laboratory staff, and outcome assessors were kept blinded also.

Interventions

The AWcorna vaccine (15 μg/dose) (batch number RR202109006) is manufactured by Yuxi Walvax Biotechnology Co. Ltd. (an affiliate of Walvax Biotechnology Co., Ltd.), and supplied in pre-filled 0.5 ml syringes. The CoronaVac (Sinovac) vaccine, is an inactivated whole-virion

vaccine with aluminum hydroxide as the adjuvant. Each dose of CoronaVac contains 3 μg SARS CoV-2 virion in a 0.5 ml aqueous suspension for injection with 0.45 mg/ml aluminum.

Assessments

After injection, subjects received an in-site 30-minutes safety observation conducted by research staff to confirm if any immediate reactions occurred. Any adverse events (AEs) discovered or any relevant concomitant medications declared within 28 days after vaccination were recorded by subjects with the help of the daily cards and connections cards which were prepared and managed by the study team. For each subject, serious adverse events (SAEs), adverse events of interest (AESI), and pregnancy were collected from the enrollment till 12 months after his/her booster dose. Up to 6 ml of blood sample was collected from each participant at baseline pre-booster vaccination and on day 14 and day 28 after receiving the booster dose.

Endpoints

The primary endpoints for safety were: 1) The incidence rates of adverse reactions/adverse events within 30 minutes, during Day 0-14 and Day 0-28 after vaccination; and 2) The incidence rates of adverse reactions/adverse events with a severity of grade 3 and above within 30 minutes and during Day 0-14 and Day 0-28 after vaccination. The primary immunogenicity endpoint was the titers of neutralizing antibody against wild type SARS-CoV-2 as measured by live virus neutralization assay 14 days post booster.

Laboratory assays

The neutralizing antibodies in sera against the wild-type strain (GenBank: MT123291), Delta variant (IQTC-IM2175251), and Omicron variant (IQTC-Y216017) (Guangzhou Customs Technology Center, Guangzhou, China) were determined by using a cytopathic effect (CPE)-based microneutralization assay. Two-fold serial dilutions (starting from 1:4) of heat-inactivated sera were tested in duplicate wells for the presence of neutralizing antibodies in the monolayer of Vero E6 cells. 100 $TCID_{50}$ of virus in 50 μl/well was incubated with 50 μl of serum in 96-well plates for 2 h. Vero E6 cells were trypsinized and resuspended in Dulbecco's Modified Eagle Medium (DMEM) containing 4% of fetal bovine serum and 1% of pen/strep at a concentration of 1.2×10^5 cells/ml and 100 μl of cells suspension were then added into the 96-well plates, followed by incubation at 37 °C, 5% CO_2 for 4 days. The neutralization was determined by the appearance of CPE in images captured with Celigo Image Cytometer on day 4 post-infection. The neutralizing antibody titer was defined as the reciprocal of the highest sample dilution that protected at least 50% of cells from CPE.

RBD-specific ELISA antibody responses were measured using an indirect ELISA assay with a cutoff titer of 1:10. The commercial Anti-SARS-CoV-2 RBD IgG ELISA kit was used for detection. Measurement was performed using a Multiskan GO reader (Thermo Fisher) to detect optical density at 450 and 630 nm using SkanIt Software for Microplate Readers (version 4.1.0.43).

The WHO international standard for anti-SARS-CoV-2 IgG (NIBSC code 20/136) was used as

a reference with the serum samples measured in this study for calibration of the serological assays. The WHO reference (NIBSC code: 20/136) is equivalent to a live viral neutralizing antibody titer of 1:139 against wild-type SARS-CoV-2 and a titer of 1:213 against the Delta variant B.1.617.2, while the WHO reference (1,000 BAU/ml in serum) is equivalent to an RBD-specific IgG ELISA antibody titer of 1:5,490. Live viral neutralizing antibodies against wild-type strain and the Delta variant and levels of RBD-binding IgG isotypes in serum were measured on days 0, 14, and 28 after the booster. Live viral neutralizing antibodies against the Omicron variant BA.1.1 were detected only on day 28 after the booster in a subgroup randomly selected from both groups.

Sample size

The sample size was determined based on the hypothesis that the booster vaccination of mRNA vaccine following the two-dose inactivated vaccine regimen be non-inferior to that of the booster of inactivated vaccine in neutralizing antibody. It was assumed that the pooled standard deviation of log_{10}-transformed neutralizing data was 0.5 and equal GMT in both the mRNA vaccine group and CoronaVac group. 200 participants in the mRNA group and 100 participants in the CoronaVac group could have at least 80% power to observe that the lower limit of the 95% confidence interval of GMT ratio between the two groups was greater than the non-inferiority margin (2/3), with the one-sided significance level of 2.5%.

Statistical analysis

The geometric mean titer (GMT) and 95% confidence interval (CI) were used to describe

neutralizing results in the mRNA vaccine group and CoronaVac group after booster vaccination, and the GMT ratio between the two groups and 95% CI were estimated. The non-inferiority result would be concluded if the lower bound of 95%CI was larger than 2/3. When the non-inferiority conclusion was concluded, the superiority would be considered sequentially if the lower bound was larger than 1. The seroconversion rate and Clopper-Pearson 95%CI after booster vaccination were estimated as well, and the difference between the two groups was calculated using the Miettinen-Nurminen method.

We assessed the number and proportion of participants with adverse reactions 0-28 days after the booster dose. For fever, besides the NMPA standard, we also derived the oral temperature by adding 0.2℃ to the collected auxiliary temperature, and then re-graded the adverse reaction based on the FDA standard to provide more comparable results with marketed vaccines. We used the $\chi 2$ test or Fisher's exact test to analyze categorical data, the t-test to analyze the log-transformed antibody titers, and the Wilcoxon rank-sum test for data not following a normal distribution. The correlation between concentrations of log-transformed neutralizing antibody and binding antibody levels was analyzed using Pearson's correlation. The primary analysis was performed based on the per-protocol population. Statistical analyses were performed using SAS (version 9.4).

Supplementary Figures

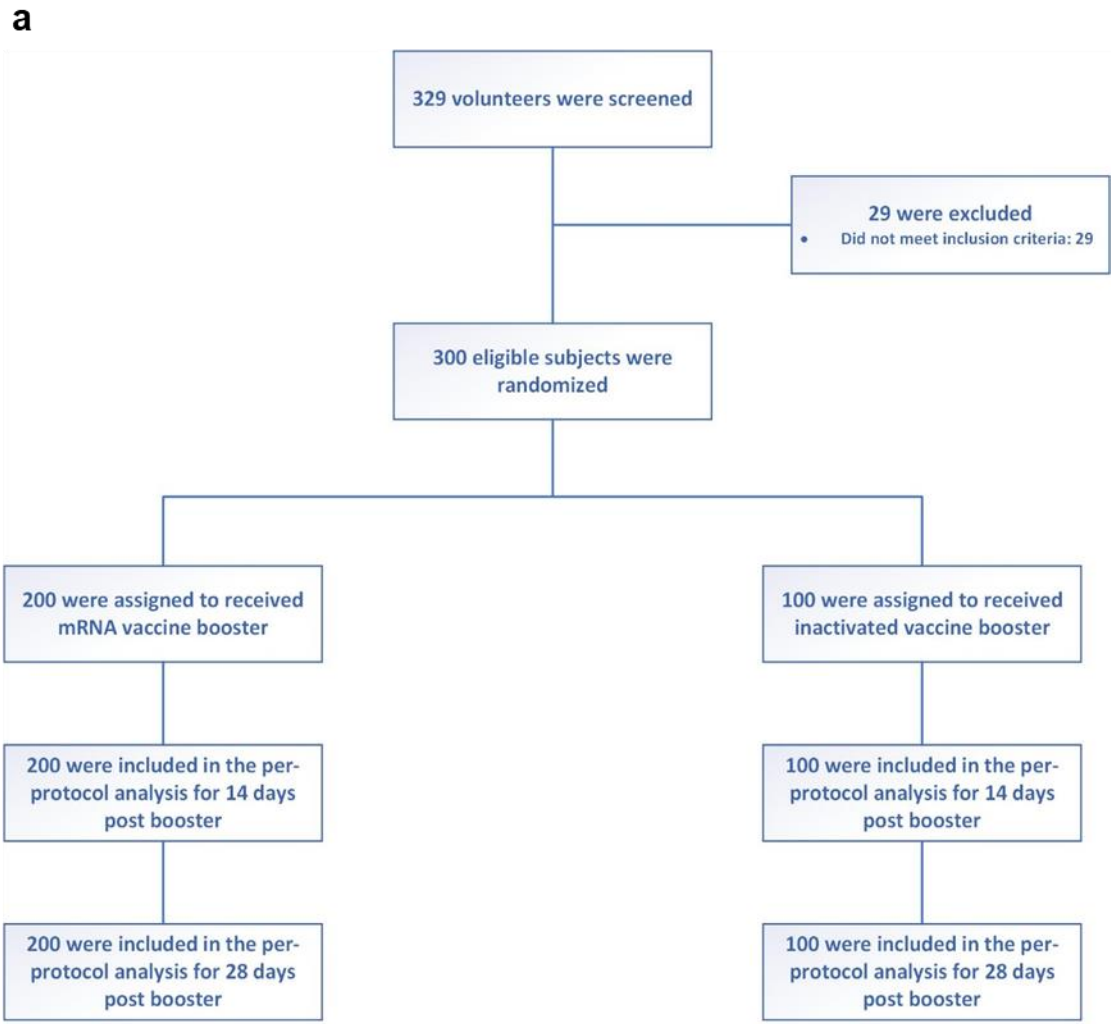

Fig. S1 Consolidated Standards of Reporting Trials (CONSORT) flow diagram

a Between the trial screening and randomization, 29 volunteers were excluded. All of them triggered the exclusion criteria. A total of 300 eligible subjects, who had received 2 doses of inactivated vaccine about 6 months ago, were randomly assigned to either the AWcorna (n=200; heterologous) or CoronaVac (n=100; homologous) booster group. All the randomized participants received vaccines at their free will and received all the scheduled follow-ups.

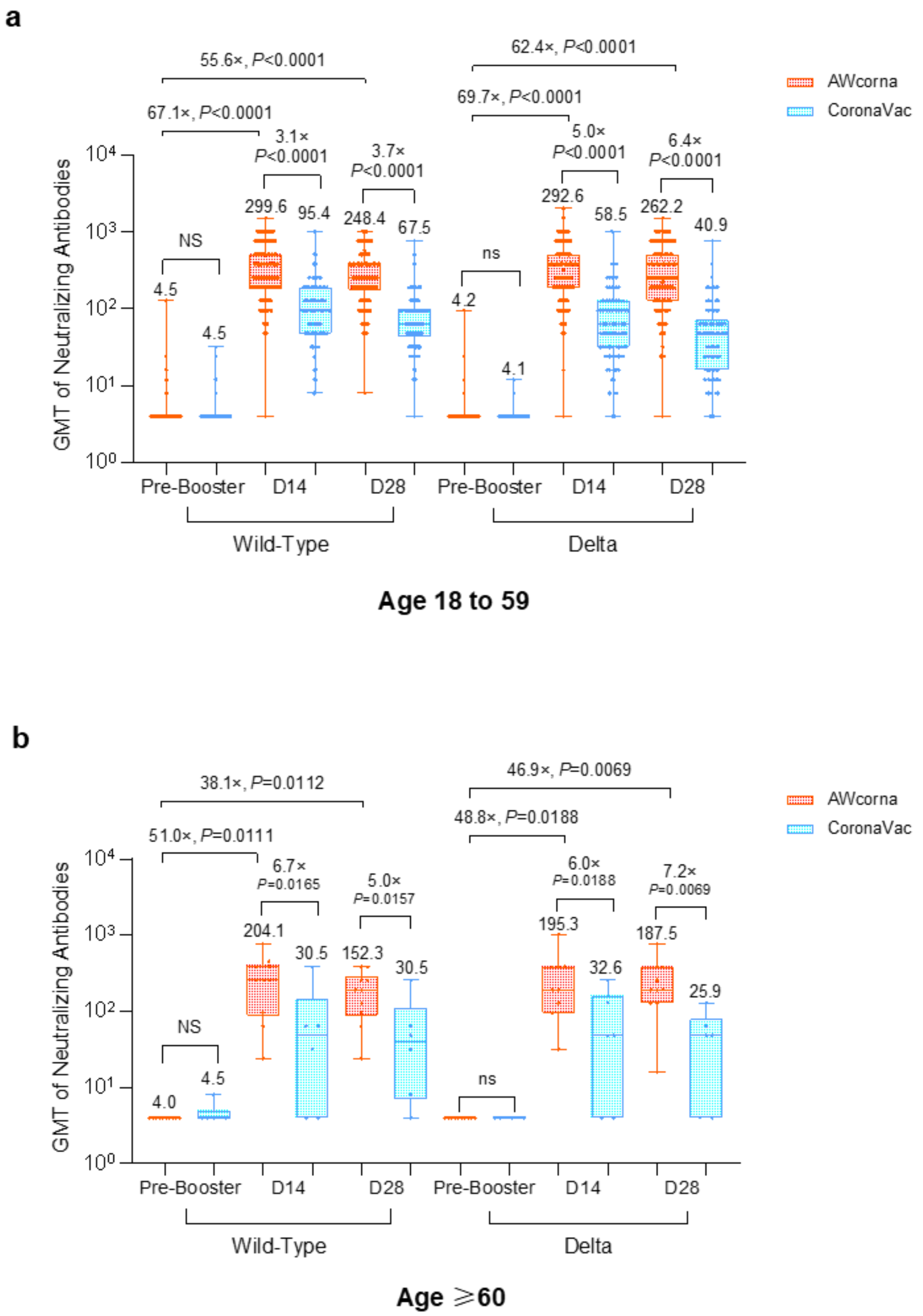

Fig.S2 Cross neutralization against WT and Delta viruses in different age groups.

a,b GMT of neutralizing antibodies to wild-type SARS-CoV-2 and Delta variant for younger adult population aged 18-59 (AWcorna=190, CoronaVac=90) (**a**) and elder population aged 60 and above (AWcorna=10, CoronaVac=6) (**b**). GMT data are presented in box-and-whisker plots. The whiskers indicate the range, the top and bottom of the boxes indicate the interquartile range, and the horizontal line within each box indicates the median. Figures with the x suffix indicate the GMT ratio between groups or GMT increase fold raised from pre- to post-booster level. *P* values were obtained from comparisons between the two treatment groups using *t*-tests for log-transformed antibody.

Table S1. Baseline characteristics of the trial participants, who had received two doses of priming inactivated vaccine. *

	AWcorna (N=200)	CoronaVac (N=100)	*P*-Value
**Age(Median, IQR)**	43.0 (36.5,49.0)	40.0 (34.0,48.5)	0.5165
**Sex, male(%)**	116 (58.00)	55 (55.00)	0.6208
**BMI(min-max)** †	23.56 (15.58-34.53)	23.22 (18.31-33.31)	0.3998
**Ethnicity**‡			
Lahu, n(%)	179 (89.50)	88 (88.00)	0.5418
Wa, n(%)	1 (0.50)	0 (0.00)	
Hani, n(%)	10 (5.00)	3 (3.00)	
Yi, n(%)	2 (1.00)	1 (1.00)	
Dai, n(%)	0 (0.00)	0 (0.00)	
Other, n(%)	8 (4.00)	8 (8.00)	
**RT-PCR, negative(%)**	200(100)	100(100)	1.0000
**Vital Signs**			
Systolic Pressure	118(81-141)	118(85-140)	0.9615
Diastolic Pressure	73(48- 89)	74(56-90)	0.3679
Pulse	79(56-114)	81(53-110)	0.2182
**Comorbidities**			
**Any (%)**	9	9	1.0000
Gastrointestinal disorders (%)	3.5	2	0.7227
Vascular disorders (%)	1	2	0.6030
Respiratory, thoracic and mediastinal disorders (%)	1.5	0	0.5533
Infections and infestations (%)	1	1	1.0000
Musculoskeletal and connective tissue disorders (%)	0.5	1	1.0000
Nervous system disorders (%)	0.5	1	1.0000
Blood and lymphatic system disorders (%)	1	0	0.5541
Endocrine disorders (%)	0	1	0.3333
Investigations (%)	0.5	0	1.0000
Reproductive system and breast disorders (%)	0	1	0.3333
**SARS-CoV-2 specific antibody titers on day 0§**			
Geometric Mean Titer of anti-RBD IgG (Mean, 95%CI)			
	134.7 (118.3, 153.4)	140.8 (115.3, 172.0)	0.7061
Geometric Mean Titer of nAb against Wild-Type SARS-CoV-2 (Mean, 95%CI)			
	4.4(4.2, 4.7)	4.5(4.2, 4.9)	0.7831
Geometric Mean Titer of nAb against Delta Variant (Mean, 95%CI)			
	4.2(4.0, 4.4)	4.1(4.0, 4.3)	0.5951

* The first trial visit occurred before the booster, and the second and third trial visit occurred 14- and 28-days after administration of the booster. IQR denotes interquartile range.

† The body-mass index (BMI) is the weight in kilograms divided by the square of the height in meters.

‡ Categories were reported by the participants. The categories shown are those used by the investigators to denote

ethnicity.

§ In the two groups, blood was drawn on day 0, which was the day of the first trial visit. Day 0 was also the day of the booster in the two groups.

Table S2. Neutralizing antibodies to the wild-type SARS-CoV-2 as well as Delta and Omicron variants before and after booster

	AWcorna N=200	CoronaVac N=100	GMT/GMI Ratio (AWcorna /CoronaVac) or Diff. in % (AWcorna -CoronaVac)	Statistics	*P* value
**Wild-Type**					
Pre-Booster					
GMT	4.4(4.2, 4.7)	4.5(4.2, 4.9)	NA	t=-0.275	0.7831
Seropositive* (%)	9.00(5.42, 13.85)	10.00(4.90, 17.62)	NA	χ^2=0.079	0.7790
D14					
GMT	293.9(260.4, 331.7)	89.1(73.7, 107.8)	3.3(2.7, 4.1)	NA	<0.0001
GMI	66.2(58.3, 75.1)	19.8(16.3, 24.0)	3.3(2.7, 4.2)	NA	<0.0001
Seroconversion* (%)	99.00(96.43, 99.88)	98.00(92.96, 99.76)	1.00(-1.92, 6.08)	NA	0.6030
4-Fold Increase (%)	99.00(96.43, 99.88)	95.00(88.72, 98.36)	4.00(0.37, 10.27)	NA	0.0433
D28					
GMT	242.4(216.4, 271.4)	64.3(53.6, 77.2)	3.8(3.1, 4.6)	NA	<0.0001
GMI	54.6(48.4, 61.6)	14.3(11.9, 17.1)	3.8(3.1, 4.7)	NA	<0.0001
Seroconversion**(%)	99.50(97.25, 99.99)	98.00(92.96, 99.76)	1.50(-1.08, 6.55)	NA	0.2585
4-Fold Increase (%)	99.00(96.43, 99.88)	94.00(87.40, 97.77)	5.00(1.11, 11.57)	NA	0.0183
**Delta**					
Pre-Booster					
GMT	4.2(4.0, 4.4)	4.1(4.0, 4.3)	NA	t=0.532	0.5951
Seropositive* (%)	3.00(1.11, 6.42)	3.00(0.62, 8.52)	NA	NA	1.0000
D14					
GMT	286.8(252.9, 325.3)	56.5(45.6, 70.0)	5.1(4.0, 6.4)	NA	<0.0001
GMI	68.5(60.1, 78.0)	13.7(11.1, 17.0)	5.0(3.9, 6.3)	NA	<0.0001
Seroconversion	99.00(96.43, 99.88)	96.00(90.07, 98.90)	3.00(-0.38, 8.93)	NA	0.0979

4-Fold Increase (%)	99.00(96.43, 99.88)	91.00(83.60, 95.80)	8.00(3.35, 15.32)	NA	0.0011
D28					
GMT	257.8(225.7, 294.5)	39.8(32.1, 49.3)	6.5(5.1, 8.2)	NA	<0.0001
GMI	61.5(53.7, 70.5)	9.7(7.8, 11.9)	6.4(5.0, 8.1)	NA	<0.0001
Seroconversion[*] (%)	99.00(96.43, 99.88)	95.00(88.72, 98.36)	4.00(0.37, 10.27)	NA	0.0433
4-Fold Increase (%)	99.00(96.43, 99.88)	81.00(71.93, 88.16)	18.00(11.27, 26.87)	NA	<0.0001
**Omicron (For Subgroup)**	**N=80**	**N=40**			
D28					
GMT	28.1(21.4, 36.8)	6.4(5.0, 8.3)	4.4(2.9, 6.7)	NA	<0.0001
Seropositive[*] (%)	83.75(73.82, 91.05)	35.00(20.63, 51.68)	48.75(30.82, 63.84)	NA	<0.0001

The geometric mean of titer (GMT) and geometric mean of increase against the pre-booster level (GMI) are presented with corresponding 2-sided 95% confidence interval (CI) respectively in AWcorna and CoronaVac groups, and the ratios of GMT or GMI between the two groups and corresponding 95% CI are accordingly showed.

The non-inferiority result would be concluded if the lower bound of 95% CI of the ratio between groups (AWcorna/CoronaVac) is larger than 2/3 When the non-inferiority conclusion is concluded, the superiority would be considered sequentially if the lower bound was larger than 1.

The seroconversion rate and the 4-fold Increase rate with their corresponding Clopper-Pearson 95% CI are shown, and the difference between the two groups were calculated using Miettinen-Nurminen method.

N=the number of participants included the per-protocol population. The p values are the results of comparison between the AWcorna and CoronaVac groups.

*Seropositive defined as the status that the nAb level of a subject achieved the threshold of 1:8 (Limit of Detection, LOD).

**Seroconversion due to booster dose at a subject level is defined as a change from below the LOD to equal or above LOD, or for those who above the LOD pre-booster experienced at least a 4-fold rise in terms of nAbs.

Table S3. RBD-Specific antibodies before and after booster

	AWcorna<br>N=200	CoronaVac<br>N=100	GMT/GMI Ratio (AWcorna /CoronaVac)<br>or Diff. in % (AWcorna -CoronaVac)	Statistics	*P* value
Pre-Booster					
GMT	134.7(118.3, 153.4)	140.8(115.3, 172.0)	NA	*t*=-0.378	0.7061
Seropositive*(%)	100.00(98.17, 100.00)	100.00(96.38, 100.00)	NA	NA	1.0000
D14					
GMT	29270.4(26074.5, 32858.1)	4317.8(3680.1, 5065.9)	6.8 (5.6, 8.3)	NA	<0.0001
GMI	217.3(184.9, 255.3)	30.7(25.5, 36.9)	7.1 (5.5, 9.2)	NA	<0.0001
Seroconversion*(%)	99.50(97.25, 99.99)	98.00(92.96, 99.76)	1.50(-1.08, 6.55)	NA	0.2585
4-Fold Increase(%)	99.50(97.25, 99.99)	98.00(92.96, 99.76)	1.50(-1.08, 6.55)	NA	0.2585
D28					
GMT	21874.4(19568.4, 24452.3)	3102.3(2661.8, 3615.8)	7.1 (5.8, 8.5)	NA	<0.0001
GMI	162.4(138.0, 191.1)	22.0(18.4, 26.4)	7.4 (5.7, 9.6)	NA	<0.0001
Seroconversion*(%)	99.00(96.43, 99.88)	95.00(88.72, 98.36)	4.00(0.37, 10.27)	NA	0.0433
4-Fold Increase(%)	99.00(96.43, 99.88)	95.00(88.72, 98.36)	4.00(0.37, 10.27)	NA	0.0433

The geometric mean of titer (GMT) and geometric mean of increase against the pre-booster level (GMI) are presented with corresponding 2-sided 95% confidence interval (CI) respectively in AWcorna and CoronaVac groups, and the ratios of GMT or GMI between the two groups and corresponding 95% CI are accordingly showed.

The seroconversion rate and the 4-fold Increase rate with their corresponding Clopper-Pearson 95% CI are shown, and the difference between the two groups were calculated using Miettinen-Nurminen method.

N=the number of subjects included the per-protocol population. The p values are the results of comparison between the AWcorna and CoronaVac groups.

*Seropositive defined as the status that the nAb level of a subject achieved the threshold of 1:10 (Limit of Detection, LOD).

** Seroconversion due to booster dose at a subject level is defined as a change from below the LOD to equal or above LOD, or for those who above the LOD pre-booster experienced at least a 4-fold rise in terms of nAbs.

Table S4. Solicited and unsolicited adverse reactions that occurred within 28 days after booster vaccination.

Event Name<br>Severity	AWcorna **(N=200)**<br>**n(%)**	**CoronaVac (N=100)**<br>**n(%)**	***P*-Value**
**AE (Any)**	139(69.5)	20(20)	**<0.0001**
Grade1	85(42.5)	12(12)	**<0.0001**
Grade2	71(35.5)	6(6)	**<0.0001**
Grade3	22(11)	3(3)	**0.0246**
Grade4	0(0)	0(0)	1
Grade5	0(0)	0(0)	1
≥Grade2	84(42)	9(9)	**<0.0001**
≥Grade3	22(11)	3(3)	**0.0246**
Solicited AE (Any)	138(69)	20(20)	**<0.0001**
Grade1	85(42.5)	12(12)	**<0.0001**
Grade2	69(34.5)	6(6)	**<0.0001**
Grade3	22(11)	3(3)	**0.0246**
Grade4	0(0)	0(0)	1
≥Grade2	83(41.5)	9(9)	**<0.0001**
≥Grade3	22(11)	3(3)	**0.0246**
Systemic (Any)	131(65.5)	20(20)	**<0.0001**
Grade1	73(36.5)	12(12)	**<0.0001**
Grade2	67(33.5)	6(6)	**<0.0001**
Grade3	20(10)	3(3)	**0.0372**
Grade4	0(0)	0(0)	1
≥Grade2	82(41)	9(9)	**<0.0001**
≥Grade3	20(10)	3(3)	**0.0372**
Fever (FDA Standard, Any)	67(33.5)	4(4)	**<0.0001**
Grade1	39(19.5)	2(2)	**<0.0001**
Grade2	20(10)	2(2)	**0.0100**
Grade3	8(4)	0(0)	0.0555
Grade4	0(0)	0(0)	1
≥Grade2	28(14)	2(2)	**0.0008**
≥Grade3	8(4)	0(0)	0.0555
Diarrhea (Any)	0(0)	0(0)	1
Grade1	0(0)	0(0)	1
Grade2	0(0)	0(0)	1
Grade3	0(0)	0(0)	1
Grade4	0(0)	0(0)	1
≥Grade2	0(0)	0(0)	1
≥Grade3	0(0)	0(0)	1
Nausea (Any)	5(2.5)	1(1)	0.6674
Grade1	4(2)	0(0)	0.3052
Grade2	1(0.5)	0(0)	1
Grade3	0(0)	1(1)	0.3333

Grade4	0(0)	0(0)	1
≥Grade2	1(0.5)	1(1)	1
≥Grade3	0(0)	1(1)	0.3333
Vomiting (Any)	5(2.5)	1(1)	0.6674
Grade1	5(2.5)	0(0)	0.1734
Grade2	0(0)	0(0)	1
Grade3	0(0)	1(1)	0.3333
Grade4	0(0)	0(0)	1
≥Grade2	0(0)	1(1)	0.3333
≥Grade3	0(0)	1(1)	0.3333
Headache (Any)	52(26)	7(7)	**<0.0001**
Grade1	11(5.5)	3(3)	0.3993
Grade2	41(20.5)	3(3)	**<0.0001**
Grade3	0(0)	1(1)	0.3333
Grade4	0(0)	0(0)	1
≥Grade2	41(20.5)	4(4)	<0.0001
≥Grade3	0(0)	1(1)	0.3333
Muscle Aches (Any)	15(7.5)	1(1)	**0.0255**
Grade1	5(2.5)	0(0)	0.1734
Grade2	9(4.5)	1(1)	0.1732
Grade3	1(0.5)	0(0)	1
Grade4	0(0)	0(0)	1
≥Grade2	10(5)	1(1)	0.1073
≥Grade3	1(0.5)	0(0)	1
Joint Pain (Any)	6(3)	1(1)	0.4310
Grade1	3(1.5)	0(0)	0.5533
Grade2	2(1)	1(1)	1
Grade3	1(0.5)	0(0)	1
Grade4	0(0)	0(0)	1
≥Grade2	3(1.5)	1(1)	1
≥Grade3	1(0.5)	0(0)	1
Chills (Any)	10(5)	1(1)	0.1073
Grade1	7(3.5)	1(1)	0.2765
Grade2	3(1.5)	0(0)	0.5533
Grade3	0(0)	0(0)	1
Grade4	0(0)	0(0)	1
≥Grade2	3(1.5)	0(0)	0.5533
≥Grade3	0(0)	0(0)	1
Fatigue (Any)	4(2)	0(0)	0.3052
Grade1	2(1)	0(0)	0.5541
Grade2	1(0.5)	0(0)	1
Grade3	1(0.5)	0(0)	1
Grade4	0(0)	0(0)	1
≥Grade2	2(1)	0(0)	0.5541

≥Grade3	1(0.5)	0(0)	1
Rash (Not at Injection Site, Any)	0(0)	1(1)	0.3333
Grade1	0(0)	0(0)	1
Grade2	0(0)	0(0)	1
Grade3	0(0)	1(1)	0.3333
Grade4	0(0)	0(0)	1
≥Grade2	0(0)	1(1)	0.3333
≥Grade3	0(0)	1(1)	0.3333
Hypersensitivity (Any)	1(0.5)	0(0)	1
Grade1	0(0)	0(0)	1
Grade2	1(0.5)	0(0)	1
Grade3	0(0)	0(0)	1
Grade4	0(0)	0(0)	1
≥Grade2	1(0.5)	0(0)	1
≥Grade3	0(0)	0(0)	1
Local AE (Any)	34(17)	2(2)	**<0.0001**
Grade1	26(13)	2(2)	**0.0013**
Grade2	7(3.5)	0(0)	0.0998
Grade3	2(1)	0(0)	0.5541
Grade4	0(0)	0(0)	1
≥Grade2	9(4.5)	0(0)	**0.0319**
≥Grade3	2(1)	0(0)	0.5541
Pain at Injection Site (Any)	34(17)	2(2)	**<0.0001**
Grade1	25(12.5)	2(2)	**0.0021**
Grade2	7(3.5)	0(0)	0.0998
Grade3	2(1)	0(0)	0.5541
Grade4	0(0)	0(0)	1
≥Grade2	9(4.5)	0(0)	**0.0319**
≥Grade3	2(1)	0(0)	0.5541
Induration (Any)	0(0)	0(0)	1
Grade1	0(0)	0(0)	1
Grade2	0(0)	0(0)	1
Grade3	0(0)	0(0)	1
Grade4	0(0)	0(0)	1
≥Grade2	0(0)	0(0)	1
≥Grade3	0(0)	0(0)	1
Redness (Any)	0(0)	0(0)	1
Grade1	0(0)	0(0)	1
Grade2	0(0)	0(0)	1
Grade3	0(0)	0(0)	1
Grade4	0(0)	0(0)	1
≥Grade2	0(0)	0(0)	1
≥Grade3	0(0)	0(0)	1

Rash (at Injection Site, Any)	0(0)	0(0)	1
Grade1	0(0)	0(0)	1
Grade2	0(0)	0(0)	1
Grade3	0(0)	0(0)	1
Grade4	0(0)	0(0)	1
≥Grade2	0(0)	0(0)	1
≥Grade3	0(0)	0(0)	1
Swelling (Any)	2(1)	0(0)	0.5541
Grade1	2(1)	0(0)	0.5541
Grade2	0(0)	0(0)	1
Grade3	0(0)	0(0)	1
Grade4	0(0)	0(0)	1
≥Grade2	0(0)	0(0)	1
≥Grade3	0(0)	0(0)	1
Itch (Any)	0(0)	0(0)	1
Grade1	0(0)	0(0)	1
Grade2	0(0)	0(0)	1
Grade3	0(0)	0(0)	1
Grade4	0(0)	0(0)	1
≥Grade2	0(0)	0(0)	1
≥Grade3	0(0)	0(0)	1
Unsolicited AE (Any)	2(1)	1(1)	1
Grade1	0(0)	0(0)	1
Grade2	2(1)	0(0)	0.5541
Grade3	0(0)	1(1)	0.3333
Grade4	0(0)	0(0)	1
Grade5	0(0)	0(0)	1
≥Grade2	2(1)	1(1)	1
≥Grade3	0(0)	1(1)	0.3333

Data are n (%). n, number of participants; %, percentage of participants; any, all participants with any grade of adverse reactions. The analysis was based on the per-protocol population. *P* values shown in bold are <0.05.
